# Paired Transcriptomic Profiling Identifies Shared Liver Metastasis-Associated Molecular Features in Colorectal Cancer

**DOI:** 10.3390/cimb48101033

**Published:** 2026-10-05

**Authors:** Hye Won Lee, Seok Soon Park, Mi Ri Kwon, Eun Jin Ju, Seol Hwa Shin, Jin Park, Eun Jung Ko, Ga Won Son, Heetaek Yang, Eun Jung Park, Seung-Jae Myung, Chan Wook Kim, Jong Lyul Lee, Jung Jin Hwang, Shin-Wha Lee, Tae Won Kim, Seong-Yun Jeong, Si Yeol Song

**Affiliations:** 1 Asan Institute for Life Sciences, Asan Medical Center, Seoul 05505, Republic of Korea; hwonxi@amc.seoul.kr (H.W.L.); wheat2@amc.seoul.kr (M.R.K.); ejju@amc.seoul.kr (E.J.J.); siseha@amc.seoul.kr (S.H.S.); jin08@amc.seoul.kr (J.P.); cdcat02@amc.seoul.kr (E.J.K.); gwhand@amc.seoul.kr (G.W.S.); yheetaek@amc.seoul.kr (H.Y.); kendoej@amc.seoul.kr (E.J.P.); sjmyung@amc.seoul.kr (S.-J.M.); crscwkim@amc.seoul.kr (C.W.K.); iamleejong@amc.seoul.kr (J.L.L.); jjhwang@amc.seoul.kr (J.J.H.); swhlee@amc.seoul.kr (S.-W.L.); twkimmd@amc.seoul.kr (T.W.K.); 2 Department of Convergence Medicine, Asan Medical Institute of Convergence Science and Technology, Asan Medical Center, University of Ulsan College of Medicine, Seoul 05505, Republic of Korea; 3 Medical Research Institute, Kangbuk Samsung Hospital, Seoul 03181, Republic of Korea; pssooni.park@samsung.com; 4 Asan Preclinical Evaluation Center for Cancer Therapeutix, Asan Medical Center, Seoul 05505, Republic of Korea; 5 Division of Colon and Rectal Surgery, Department of Surgery, Asan Medical Center, University of Ulsan College of Medicine, Seoul 05505, Republic of Korea; 6 Department of Gastroenterology, Asan Medical Center, University of Ulsan College of Medicine, Seoul 05505, Republic of Korea; 7 Department of Convergence Medicine, Asan Medical Center, University of Ulsan College of Medicine, Seoul 05505, Republic of Korea; 8 Department of Obstetrics and Gynecology, Asan Medical Center, University of Ulsan College of Medicine, Seoul 05505, Republic of Korea; 9 Department of Oncology, Asan Medical Center, University of Ulsan College of Medicine, Seoul 05505, Republic of Korea; 10 Department of Radiation Oncology, Asan Medical Center, University of Ulsan College of Medicine, Seoul 05505, Republic of Korea

**Keywords:** Colorectal cancer, liver metastasis, tumor heterogeneity, metastasis-associated genes

## Abstract

Colorectal cancer liver metastasis (CCLM) is closely associated with poor patient prognosis, yet patient-specific molecular heterogeneity complicates the identification of recurrent molecular changes during metastasis. To address this, we performed RNA sequencing on paired primary tumors and liver metastases synchronously resected from six patients and analyzed gene expression changes using a paired design that accounted for interpatient variability. This analysis identified differentially expressed genes (DEGs) and metastasis-associated pathways through functional enrichment analysis and gene set enrichment analysis (GSEA). The reproducibility of these changes was further assessed across multiple independent publicly available transcriptomic datasets from the Gene Expression Omnibus (GEO) and The Cancer Genome Atlas (TCGA). Selected candidate genes were additionally evaluated in a metastasis-selected colorectal cancer cell model, in which several genes showed expression changes consistent with the transcriptomic findings. Together, these results indicate that recurrent molecular changes accompany CCLM despite substantial interpatient heterogeneity and provide a strategy for identifying metastasis-associated molecular features and potential therapeutic targets.

## 1. Introduction

Colorectal cancer (CRC) remains the third most common malignancy worldwide, with approximately 2 million new cases diagnosed annually, making it a leading cause of cancer-related mortality [1,2]. Moreover, the 5-year survival rate decreases markedly once distant metastasis occurs [3]. Among the sites of distant dissemination, the liver is the most common target organ, and colorectal cancer liver metastasis (CCLM) represents a major cause of mortality in patients with CRC [2,4]. For patients with CCLM, surgical resection is currently the only potentially curative treatment, boasting 5-year survival rates above 50% for eligible cohorts [5]. Unfortunately, up to 80% of patients are initially ineligible for resection due to advanced disease, and even after resection, more than half of patients experience a recurrence [5,6]. Because long-term survival remains difficult to achieve without effective control of liver metastases despite advances in chemotherapy, the development of improved diagnostic and therapeutic strategies remains critically important.

Although many studies have investigated the molecular mechanisms of CCLM, the high molecular heterogeneity observed in colorectal cancer remains a significant challenge in clearly identifying the changes associated with metastasis [7]. In particular, when primary and metastatic tumors from different patients are compared independently, it is difficult to determine whether the observed differences arise from the metastatic process itself or from each patient’s unique molecular background [8]. Paired comparison of primary and metastatic tumors obtained from the same patient allows changes accompanying the metastatic process to be evaluated while accounting for baseline differences between patients, with each patient serving as their own control. The key question is whether such changes recur across patients despite substantial molecular heterogeneity.

This study focuses on distinguishing metastasis-associated transcriptomic changes from strong patient-specific molecular backgrounds in colorectal cancer. We performed RNA sequencing of synchronously resected primary colorectal tumors and matched liver metastases from six patients to identify recurrent transcriptional changes associated with metastatic progression. We also characterized the related biological processes, assessed the reproducibility of these changes across independent transcriptomic datasets, and further examined these findings in an experimental metastatic model. Our aim was to define shared molecular features of CCLM and provide insight into the molecular changes associated with metastatic progression.

## 2. Materials and Methods

### 2.1. Patient Samples and RNA Sequencing

A total of 12 tumor specimens were obtained from six patients, comprising six matched pairs of primary colorectal tumors and corresponding synchronous liver metastases. All patients provided written informed consent, and this study was approved by the Asan Medical Center Institutional Review Board (IRB No. 2023-1603). RNA sequencing and initial data processing were performed by Macrogen Inc. (Seoul, Republic of Korea). RNA sequencing was conducted on the Illumina NovaSeqX (Illumina, San Diego, CA, USA) platform using paired-end 2 × 101 bp reads, generating approximately 76–83 million processed reads per sample. Adapter sequences and low-quality bases were trimmed using Trimmomatic v0.38, and the cleaned reads were aligned to the human reference genome GRCh38 using HISAT v2.1.0. Gene annotation was based on the NCBI RefSeq database. Gene-level expression was quantified using StringTie v2.1.3b. For downstream analysis, the gene-level read count columns were extracted from the expression output table and compiled into a count matrix without additional transformation or normalization. The RNA-sequencing data generated and analyzed in this study have been deposited in the NCBI Gene Expression Omnibus (GEO) under accession number GSE347316.

### 2.2. Data Processing and Matched-Patient Transcriptomic Similarity Analysis

Raw count matrices were subjected to variance-stabilizing transformation (VST) using the DESeq2 R package [9] (version 1.48.2) to assess the transcriptomic relatedness among synchronous tumor samples via principal component analysis (PCA). Pairwise Pearson correlation coefficients were calculated, and the resulting correlation matrices were visualized using the pheatmap R package (version 1.0.13). To statistically evaluate whether matched patient pairs were more similar than unmatched patient pairs, an exact permutation test was performed using the mean Pearson correlation coefficient of the six matched pairs as the test statistic, with all 720 possible permutations evaluated. The results were visualized using the ggplot2 (version 4.0.3) and ggrepel (version 0.9.8) R packages.

### 2.3. Paired DEG and Functional Enrichment Analyses

Paired DEG analysis was conducted using the DESeq2 (version 1.48.2) R package with the design formula ~ patient + tissue. Genes with |log2FC| ≥ 1 and FDR < 0.05 were considered differentially expressed. Functional enrichment analysis of the identified DEGs was performed using Enrichr (https://maayanlab.cloud/Enrichr/, accessed on 1 September 2026), a web-based gene set enrichment analysis tool [10,11], to obtain Gene Ontology (GO) enrichment results. Metastasis-upregulated and primary-upregulated DEGs were submitted to Enrichr separately using the GO Biological Process (BP) 2026 library and the default Enrichr background. Terms with an adjusted *p*-value < 0.05 were considered statistically significant. The enriched GO biological process (BP) terms from both directions were visualized together in a single bar plot using GraphPad Prism 9 software (La Jolla, CA, USA).

### 2.4. Gene Set Enrichment Analysis (GSEA)

GSEA was performed to identify enriched Hallmark pathways associated with the metastatic transcriptional program. Hallmark gene sets for Homo sapiens were retrieved using the msigdbr (version 26.1.1) R package, and enrichment scores were calculated using the fgsea (version 1.34.2) R package. GSEA plots were assembled using the ggplot2 (version 4.0.3) and patchwork (version 1.3.2) R packages.

### 2.5. External Validation

GSE14297 (18 matched pairs, microarray), GSE49355 (13 matched pairs, microarray), and GSE50760 (18 matched pairs, RNA-seq) were retrieved from the GEO database using the GEOquery (version 2.76.0) and Biobase (2.68.0) R packages, and paired DEGs were independently identified for each dataset using the limma (version 3.64.3) R package. GSE213402 was also retrieved from the GEO database. Venn diagrams were constructed using the ggplot2 (version 4.0.3) R package. The UpSet plot was constructed using the ComplexUpset (version 1.3.3) R package and visualized using the ggplot2 (version 4.0.3) and forcats (version 1.0.1) R packages. Robust Rank Aggregation (RRA) analysis was performed using the RobustRankAggreg (version 1.2.1) R package. RRA scores were calculated using the RobustRankAggreg package.

### 2.6. Survival Analysis

GSE17536, GSE17537, GSE39582, GSE159216, and GSE39084 were retrieved from the GEO database using the GEOquery (version 2.76.0) and Biobase (2.68.0) R packages. TCGA-COAD and TCGA-READ cohorts were obtained using the TCGAbiolinks (2.36.0) R package. Univariate Cox proportional-hazards regression was performed using the survival (version 3.8.11) R package to evaluate the prognostic significance of candidate gene expression, and hazard ratios with 95% confidence intervals were extracted using the broom (version 1.0.13) R package. Forest plots were generated using the ggplot2 (version 4.0.3) R package.

### 2.7. Cell Culture and Establishment of a CRC Cell Line with High Metastatic Potential

The human colorectal adenocarcinoma cell line HT29 was obtained from the ATCC and cultured in McCoy’s 5A Medium Modified (16600-082, Gibco, Grand Island, NY, USA) supplemented with 10% FBS and 1% antibiotics. To establish a highly metastatic cell line, HT29 cells were first enriched by repeated *in vitro* Transwell selection using 8 μm pore membranes. Cells that successfully migrated across the membrane were expanded in culture, and this selection cycle was repeated three times. The resulting *in vitro*-selected population was then introduced into 6-week-old male BALB/c-nude mice (Gem biosciences, Cheongju, Korea) via intrasplenic injection, with 5 × 10^6^ cells injected per mouse. During each round of *in vivo* selection, the mice were examined at selected time points to confirm the development of liver metastases. Metastatic tumor tissues were then dissected from the liver, cultured *in vitro*, and expanded to recover viable tumor cells. The recovered cells were subsequently subjected to the same *in vivo* selection cycle three times [12]. This iterative selection process yielded a highly liver-metastatic cell line, designated metastasis-selected HT29 (HT29-M).

For comparison of metastatic potential, HT29 parental (HT29-P) cells and HT29 metastasis-selected (HT29-M) cells were separately introduced into 6-week-old male BALB/c-nude mice by intrasplenic injection. Six mice were used per group, and 5 × 10^6^ cells were injected per mouse. The mice were sacrificed 20 days after injection, and the livers were harvested for evaluation of liver metastasis. Liver images were analyzed using QuPath software. For each liver, the total liver area and the area occupied by grossly visible metastatic lesions were delineated. Liver metastatic burden was calculated as the percentage of metastatic area relative to the total liver area. These were visualized together in a single bar plot using GraphPad Prism v9.5.1 software (La Jolla, CA, USA).

All animal procedures followed a protocol approved by the Institutional Animal Care and Use Committee of the Asan Institute for Life Science (2024-20-023).

### 2.8. Cell Invasion Assay

For the invasion assay, Transwell inserts with an 8 μm pore (Corning, NY, USA) were coated with Matrigel (Corning, NY, USA) at 150 μg in 100 μL per insert and incubated at 37 °C for 30 min. After incubation, the remaining Matrigel solution was removed while the coated layer was retained on the membrane. Cells were seeded into the upper chamber at 3 × 10^4^ cells in 100 μL of medium containing 10% FBS, and 600 μL of medium containing 10% FBS was added to the lower chamber. After 24 h, the medium in the upper chamber was replaced with 100 μL of medium containing 0.1% FBS, whereas the lower chamber was maintained with 600 μL of medium containing 10% FBS. After an additional 48 h of incubation, non-invasive cells remaining on the upper surface of the membrane were gently removed with a cotton swab, and the inserts were washed with PBS. Cells that had invaded through the membrane were fixed and stained with a solution containing 0.5% crystal violet and 20% methanol for 30 min with gentle shaking. The inserts were then washed with distilled water (DW), air-dried, and examined using a BX53 microscope (Olympus, Tokyo, Japan). Multiple microscopic fields were imaged from each insert, and the stained area corresponding to invaded cells was quantified using ImageJ 1.54g with the same threshold settings applied to all images.

### 2.9. Quantitative Real-Time Polymerase Chain Reaction (qPCR) Analysis

Total RNA was extracted from HT29-P and HT29-M cells using the RNeasy mini kit (Qiagen, Venlo, The Netherlands). Real-time PCR was performed using SYBR Green master mix (Thermo Fisher Scientific, Waltham, MA, USA) according to the manufacturer’s instructions, and fluorescence signals were measured using an Applied Biosystems StepOnePlus™ system (Thermo Fisher Scientific, Waltham, MA, USA).

Primer pairs were as follows: APOC1-forward, 5′-CTGAGTGGGGAAAGGGACTA-3′; APOC1-reverse, 5′-GGAGGGGCACTCTCTCAATC-3′; APOE-forward, 5′-TAAGCTTGGCACGGCTGTCCAAGGA-3′; APOE-reverse, 5′-ACAGAATTCGCCCCGGCCTGGTACAC-3′; COLEC11-forward, 5′-GCGTGCTCGGTCCAGATCCTC-3′; COLEC11-reverse, 5′-CTTCCCGTGATGTCCAGTGCTA-3′; CYP1B1-forward, 5′-ACGTACCGGCCACTATCACT-3′; CYP1B1-reverse, 5′-CTCCCCACGACCTGATCCA-3′; FMO3-forward, 5′-CCCACATGCTTTGAGAGGAG-3′; FMO3-reverse, 5′-GGAAGAGTTGGTGAAGACCG-3′; SPP1-forward, 5′-CGAGGTGATAGTGTGGTTTATGG-3′; SPP1-reverse, 5′-GCACCATTCAACTCCTCGCTTTC-3′; β-actin-forward, 5′-GGAGTCCTGTGGCATCCACG-3′; β-actin-reverse, 5′-CTAGAAGCATTTGCGGTGGA-3′.

### 2.10. Statistical Analysis

Statistical analyses were conducted using R software (version 4.5.1) and GraphPad Prism v9.5.1 (La Jolla, CA, USA). Mean values were compared using two-tailed *t*-tests or analysis of variance with Bonferroni correction, as appropriate. Statistical significance was defined as an adjusted *p* value (FDR) < 0.05 (*, *p* < 0.05; **, *p* < 0.01; ***, *p* < 0.001; ****, *p* < 0.0001).

## 3. Results

### 3.1. Transcriptome Profiling and Overall Characterization of Primary Tumor–Liver Metastasis Pairs

**Figure 1:**
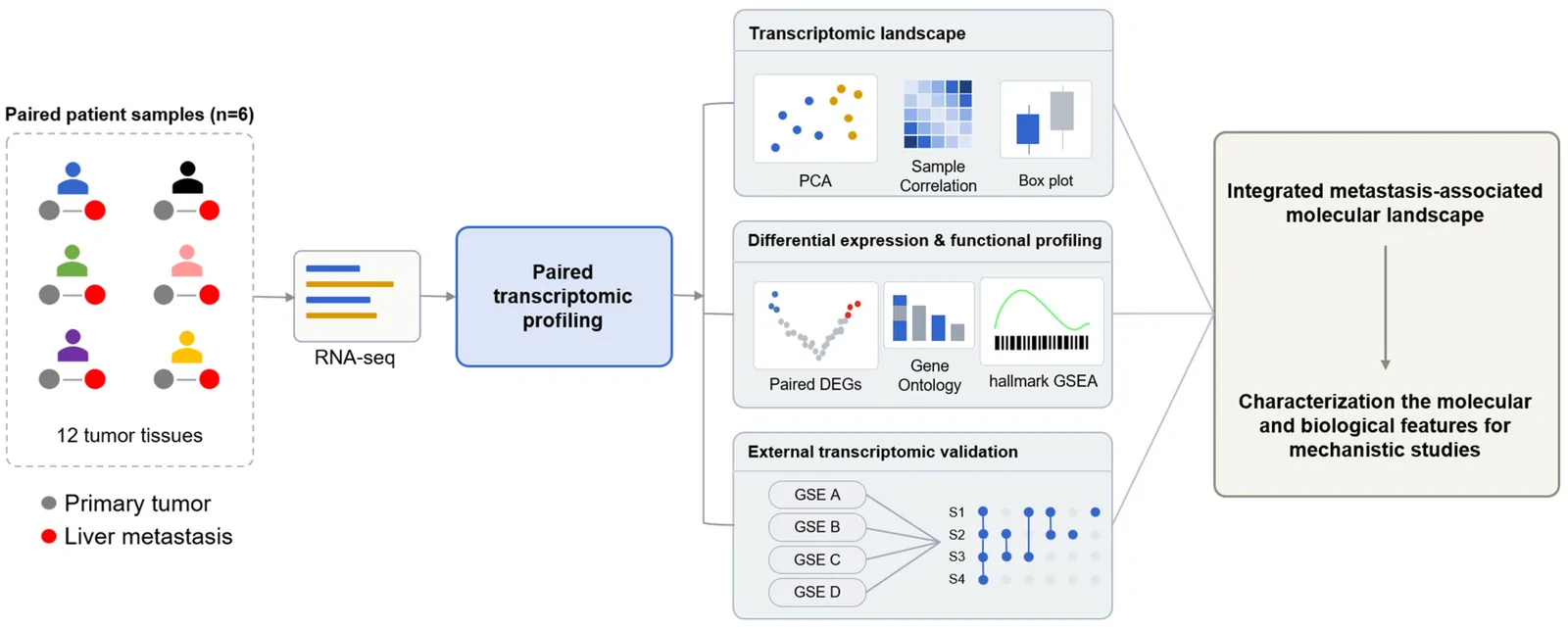
Overview of the study design and analytical workflow. Paired primary colorectal tumors and synchronous liver metastases were obtained from six patients and subjected to RNA sequencing. Transcriptomic profiles were analyzed by PCA, sample correlation, paired DEGs, GO enrichment, and GSEA, followed by validation in independent transcriptomic datasets.

We performed RNA sequencing on a total of 12 tissue samples obtained from six patients with colorectal cancer to characterize gene expression alternations associated with the process of liver metastasis. The clinicopathological characteristics of the study cohort are summarized in Table S1. The study was designed around paired profiling that directly compared the primary tumor and liver metastasis from the same patient, followed by analyses of gene expression changes and associated biological characteristics and validation using independent public datasets (Figure 1). Using this approach, we examined the differences between the primary tumor and liver metastasis from multiple perspectives and sought to identify molecular characteristics associated with colorectal cancer liver metastasis for subsequent functional investigation.

**Figure 2:**
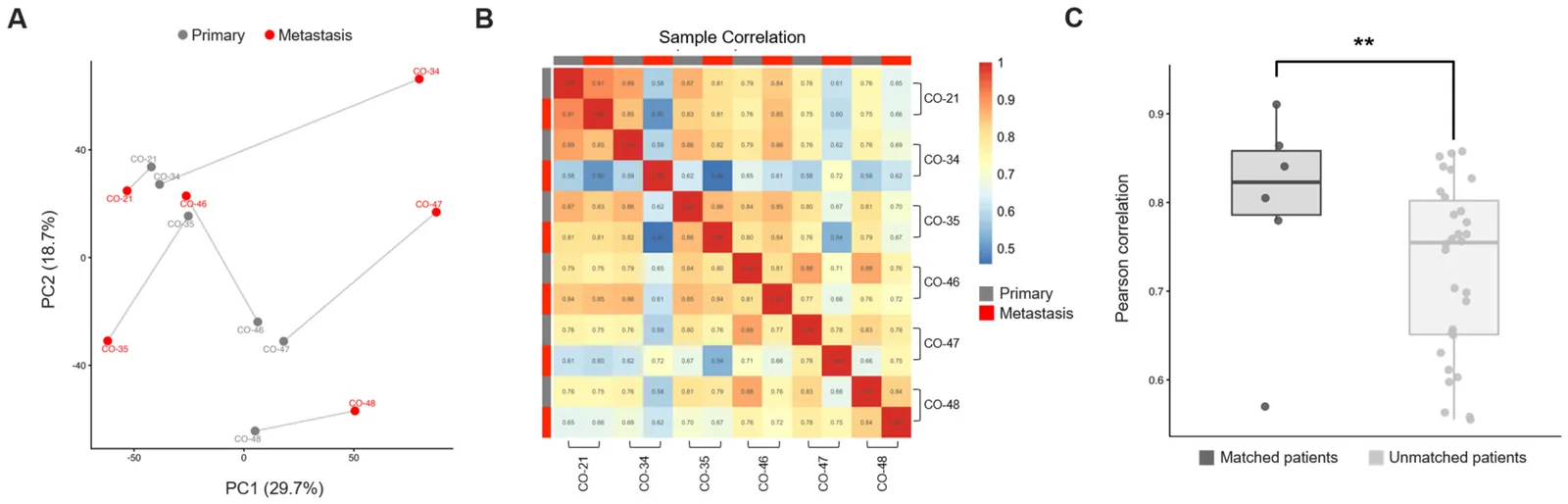
Transcriptomic landscape of paired primary colorectal tumors and liver metastases. (**A**) PCA plot from six paired primary tumors and liver metastases. Primary and metastatic samples from the single patient are connected by lines. (**B**) Correlation matrix showing Pearson correlation coefficients among the 12 tumor samples. (**C**) Comparison of Pearson correlation coefficients between matched primary–metastasis and unmatched primary–metastasis pairs. Individual dots represent pairwise correlation coefficients. Statistical significance was assessed using a permutation test (*p* = 0.0097). **, *p* < 0.01.

Principal component analysis (PCA) was performed to evaluate the overall transcriptomic characteristics of primary tumors and liver metastases. PC1 and PC2 explained 29.7% and 18.7% of the total variance, respectively (Figure 2A). Primary tumors and liver metastases occupied different positions across patients, and the distance between paired primary and metastatic samples also varied. We next performed Pearson correlation analysis to evaluate expression similarity between samples (Figure 2B). Some patient pairs showed relatively high similarity, whereas others showed lower similarity. Based on these results, we compared Pearson correlation coefficients between matched primary–metastasis pairs from a single patient and unmatched pairs from different patients to determine whether transcriptomic similarity differed between the two groups (Figure 2C). Matched pairs showed overall higher transcriptome correlations than unmatched pairs, and the difference was found to be significant in the permutation test. These findings support the need for analyses that take into account the transcriptomic background of each patient.

### 3.2. Identification of Changes Associated with Colorectal Cancer Liver Metastasis Through Paired Differential Expression Analysis

**Figure 3:**
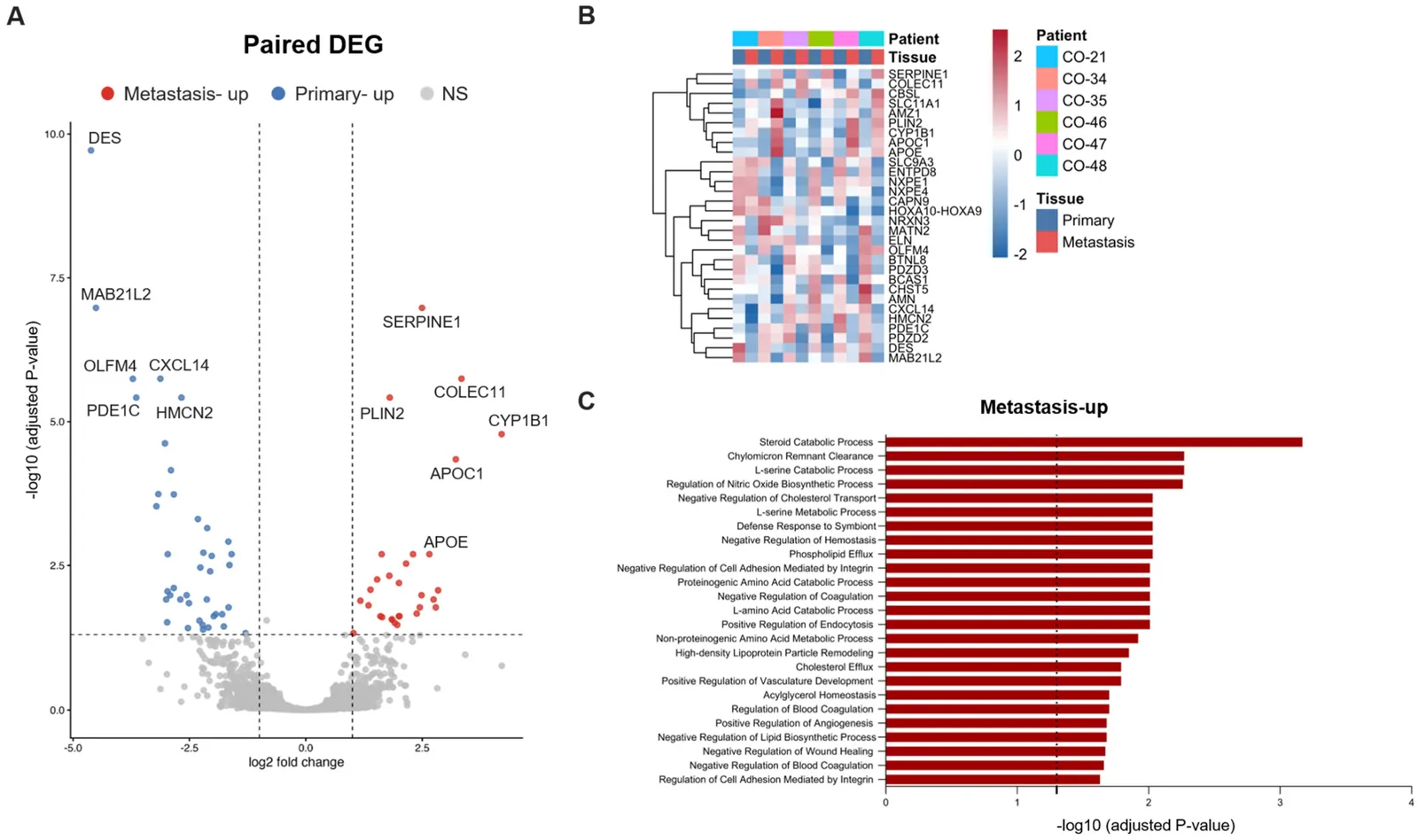
Paired differentially expressed genes (DEGs) and functional enrichment analyses of primary colorectal tumors and liver metastases. (**A**) Volcano plot showing paired DEGs between primary tumors and matched liver metastases. Genes with an absolute log2 fold change ≥ 1 and an adjusted *p* value < 0.05 were considered differentially expressed. Metastasis-up (red): metastasis-upregulated genes. Primary-up (blue): primary-upregulated genes. NS (grey): non-significant genes. (**B**) Heatmap of the top 30 DEGs. (**C**) Gene Ontology—biological process (GO-BP) enrichment analysis of genes upregulated in liver metastases. Bars represent −log10 (adjusted *p* values) for enriched biological processes; the dashed line indicates an adjusted *p* value of 0.05.

To identify gene expression changes associated with liver metastasis while accounting for interpatient heterogeneity, we performed a paired differential expression analysis. This analysis identified 71 differentially expressed genes, including 30 genes upregulated in liver metastases and 42 genes upregulated in primary tumors (Figure 3A). Genes showing significant differential expression in the volcano plot were classified as “metastasis-upregulated” and “primary-upregulated” according to the direction of change. Next, we generated a hierarchical clustering heatmap to examine the expression patterns of the top DEGs. The top 30 DEGs showed distinct expression patterns between primary tumors and liver metastases, although the magnitude of expression change varied among patients for some genes (Figure 3B). These findings demonstrate the coexistence of gene expression changes common to the metastatic process and patient-specific molecular backgrounds.

Subsequently, we performed a Gene Ontology (GO) enrichment of the DEGs. GO Biological Process analysis of metastasis-upregulated DEGs showed that the significantly enriched terms could be broadly classified into processes related to lipid/cholesterol metabolism, amino acid metabolism, vascular and hemostatic/coagulation regulation, and cell adhesion and intracellular uptake (Figure 3C). In contrast, primary-upregulated DEGs were associated with muscle/skeletal muscle and cardiac development, extracellular matrix, and cell differentiation, although these terms did not reach statistical significance (Figure S1).

### 3.3. Validation of Metastasis-Related Genes Using Publicly Available Datasets

**Figure 4:**
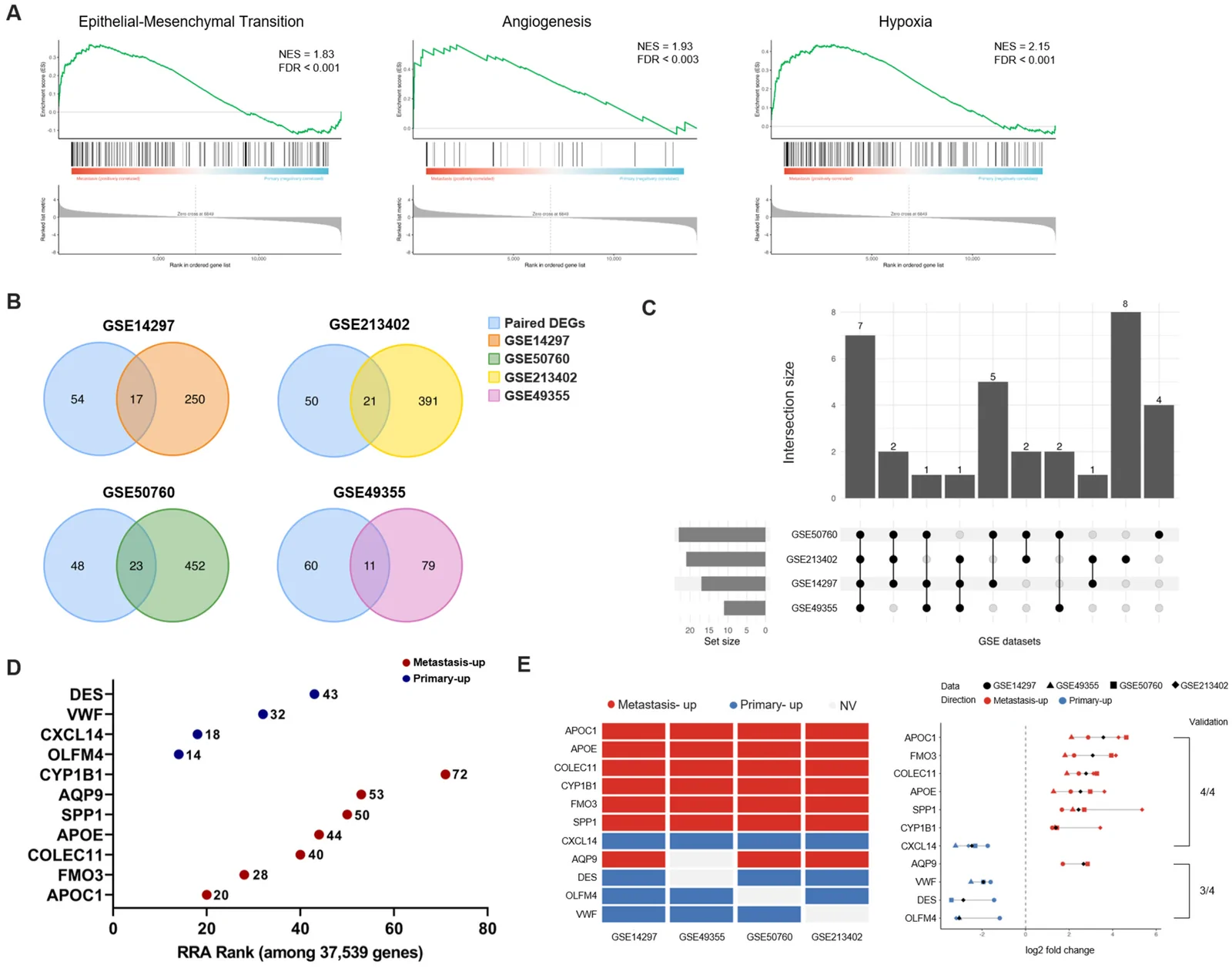
Gene set enrichment analysis (GSEA) and external validation of metastasis-associated transcriptional changes. (**A**) GSEA of a ranked gene list based on paired primary–metastasis comparison. Positive normalized enrichment scores (NESs) indicate enrichment toward liver metastases. (**B**) Venn diagrams showing the overlap between paired DEGs and DEGs identified in each of four GEO datasets. (**C**) UpSet plot summarizing intersections among overlapping DEGs identified in the paired analysis and each of the four independent GEO datasets. (**D**) Robust Rank Aggregation (RRA) analysis of the 11 prioritized candidate genes across four independent external datasets. Numbers indicate the RRA rank among 37,539 genes, with lower ranks reflecting stronger cross-cohort consistency. (**E**) Cross-cohort validation of representative metastasis-associated genes. Left, matrix showing the direction of expression change across four independent GEO datasets. Metastasis-up (red): metastasis-upregulated genes. Primary-up (blue): primary-upregulated genes. NV (grey): no validation. Right, log2 fold changes for each gene across the four GEO datasets, with each symbol representing an individual dataset.

We next used GSEA to identify biological programs associated with liver metastasis. The analysis showed that epithelial–mesenchymal transition was enriched in liver metastases (NES = 1.83, FDR q < 0.001), angiogenesis was enriched in liver metastases (NES = 1.93, FDR q < 0.003), and hypoxia was enriched in liver metastases (NES = 2.15, FDR q < 0.001) (Figure 4A). These findings indicate that metastasis-associated changes occur not only at the level of individual DEGs but also at the level of broader biological programs. This confirmed that changes exist not only at the level of individual DEGs but also at the level of biological programs associated with the metastatic process.

Next, we evaluated whether the paired DEGs were replicated in independent colorectal cancer liver metastasis cohorts. We used four external datasets: GSE14297, GSE50760, GSE213402, and GSE49355. Comparison of the DEGs identified in our study with significant genes in each external dataset showed that 17 genes overlapped with GSE14297, 23 with GSE50760, 21 with GSE213402, and 11 with GSE49355 (Figure 4B). To assess consistency across multiple independent datasets, we selected seven genes whose expression changes were confirmed in all four datasets and four genes that showed consistent changes in three of the four datasets, yielding a total of 11 candidate genes (Figure 4C). To further evaluate the robustness of this overlap-based selection, we performed Robust Rank Aggregation using gene rankings from the four external datasets. APOC1, APOE, COLEC11, CYP1B1, FMO3, SPP1, and AQP9 ranked significantly in the metastasis-upregulated direction, whereas CXCL14, DES, OLFM4, and VWF ranked significantly in the primary-upregulated direction (Figure 4D, Table S2). For these 11 metastasis-related candidate genes, we examined the direction of expression change between primary and metastatic tumors (Figure 4E). Six genes—APOC1, APOE, COLEC11, CYP1B1, FMO3, and SPP1—were consistently identified as metastasis-upregulated genes in all datasets, whereas CXCL14 was consistently identified as a primary-upregulated gene. In addition, four genes—AQP9, DES, OLFM4, and VWF—showed either metastasis-upregulated or primary-upregulated expression in three of the datasets. Analysis of fold-change values across datasets further showed consistent expression patterns for most genes. Overall, these findings indicate that the 11 candidate genes show substantial reproducibility across independent cohorts.

### 3.4. Clinical and Experimental Verification of Metastasis-Related Genes

**Figure 5:**
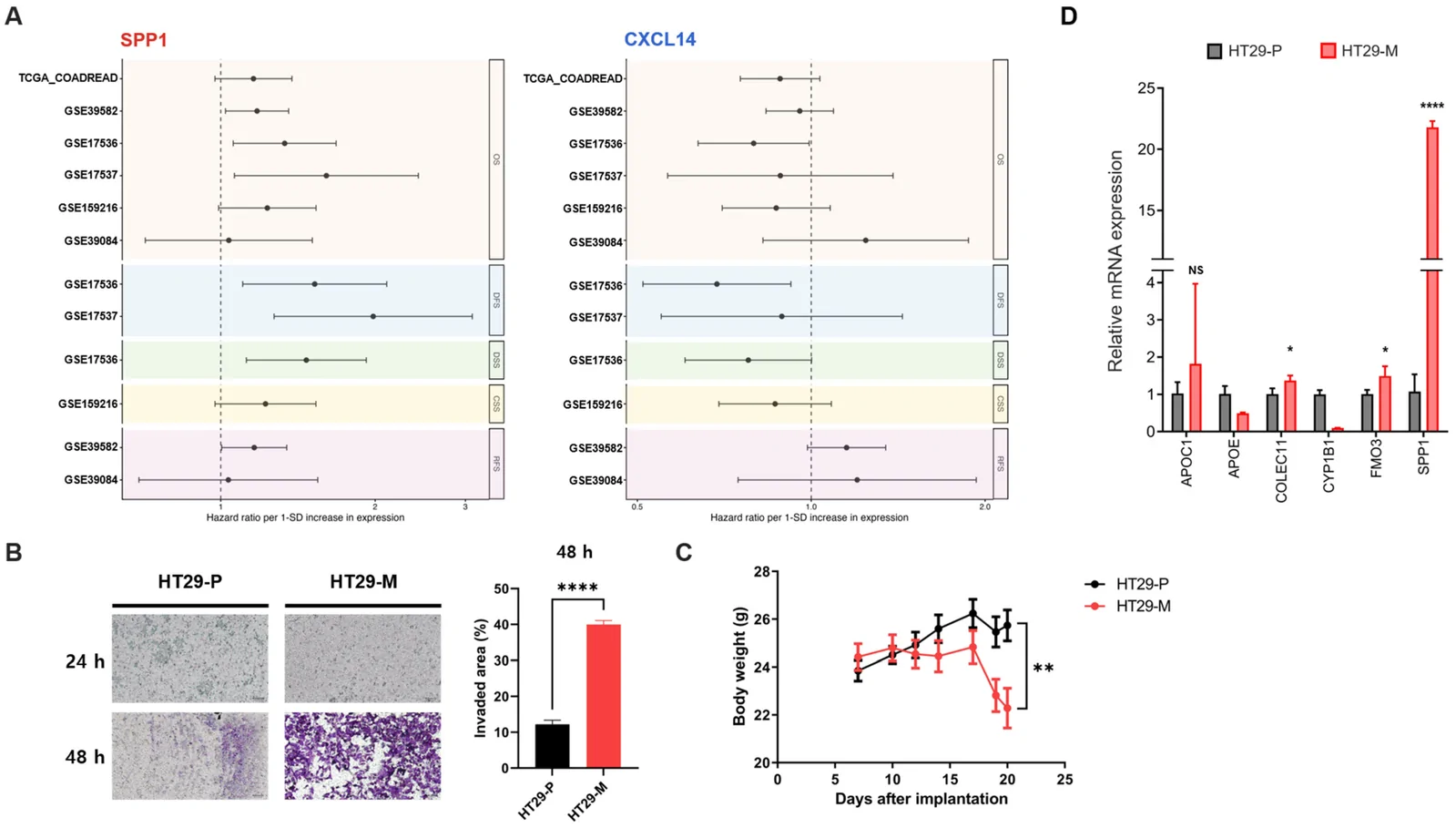
Clinical relevance and experimental validation of metastasis-associated genes. (**A**) Forest plots showing the associations between gene expression and survival outcomes across multiple datasets. SPP1 represents metastasis-upregulated genes, whereas CXCL14 represents a primary-upregulated gene. Hazard ratios (HRs) are presented per one-standard-deviation increase in gene expression, with horizontal lines indicating 95% confidence intervals. The dashed vertical line indicates HR = 1. Survival outcomes include overall survival (OS), disease-free survival (DFS), disease-specific survival (DSS), cancer-specific survival (CSS), and recurrence-free survival (RFS). The dashed vertical line indicates HR = 1. (**B**) Representative images and quantification of Transwell invasion assays comparing HT29-P and HT29-M cells. Scale bar, 50 μm. (**C**) BALB/c nude mice were intrasplenically injected with HT29-P or HT29-M cells, and body weight changes were monitored. Data are presented as the mean ± SD. Statistical significance was assessed between the two groups at the experimental endpoint. **, *p* < 0.01. (**D**) qPCR analysis of metastasis-upregulated candidate genes in HT29-P and HT29-M cells. Expression levels are shown relative to HT29-P cells. Data are presented as mean ± SD. Statistical significance was determined using an unpaired two-tailed Student’s *t*-test. NS, not significant; *, *p* < 0.05; ****, *p* < 0.0001.

Survival analysis was performed as a complementary and exploratory assessment of the clinical relevance of the prioritized metastasis-associated genes. For the seven genes identified across all four validation datasets, available survival endpoints (overall survival, DFS, DSS, CSS, and RFS) were analyzed in each cohort (Figure 5A). This analysis examines whether the expression of genes identified as metastasis-associated during the primary-to-liver-metastasis transition is also related to clinical outcome when measured in patient tumors. SPP1 showed a trend toward increased hazard ratios across multiple cohorts, although statistical significance varied. In contrast, CXCL14, a primary-upregulated gene, generally showed hazard ratios below 1 across most cohorts, although statistical significance varied among cohorts. The remaining five genes were also analyzed across independent cohorts and showed variable prognostic associations depending on the cohort and survival endpoint (Figure S2). These findings suggest that metastasis-associated expression patterns may also be reflected in primary tumors and could have potential clinical relevance.

Next, we evaluated whether the identified gene expression characteristics were reproduced in the experimental model. A sequential metastasis-selection process was performed *in vitro* and *in vivo* to establish the metastasis-selected cell line HT29-M. We conducted a Transwell assay to assess the invasion ability of HT29-M cells and confirmed that they showed increased invasive characteristics compared with the HT29-P cells (Figure 5B). In addition, an intrasplenic metastasis mouse model was established using HT29-P cells and HT29-M cells, and body weight changes were monitored. Greater body weight loss was observed in the HT29-M group (Figure 5C). Liver metastatic burden was additionally quantified as the proportion of metastatic area relative to the total liver surface area, which was significantly higher in the HT29-M group than in the HT29-P group. Liver images and quantitative analysis of metastatic surface involvement are provided in Figure S3. qPCR was performed to determine whether metastasis-related gene expression changes were recapitulated in the established cell model (Figure 5D). COLEC11, FMO3, and SPP1 showed expression changes in HT29-M cells that were consistent with those observed in the paired transcriptomic analysis and independent cohorts. APOC1 showed a similar directional trend, although the difference did not reach statistical significance. In contrast, APOE and CYP1B1 did not reproduce the expression patterns observed in the transcriptomic analysis. These results suggest that although this model does not fully recapitulate the complex biological environment of liver metastases in patients, it may still be useful for experimentally evaluating selected transcriptomic changes and metastasis-related characteristics.

## 4. Discussion

Colorectal cancer liver metastasis is characterized by marked heterogeneity among patients, and the biological and molecular changes associated with metastatic progression are complex [13,14]. These features underscore the need to account for patient-specific characteristics during analysis. When primary and metastatic tumors from different patients are compared, observed gene expression differences may arise not only from metastasis-associated changes but also from the unique molecular background of each patient. Therefore, identifying molecular characteristics associated with colorectal cancer liver metastasis requires distinguishing interpatient differences from changes that occur during the metastatic process.

To minimize the influence of interpatient heterogeneity, we analyzed paired primary colorectal tumors and liver metastases obtained from the same patients. Overall transcriptomic patterns indicated that patient-specific molecular characteristics remained prominent across primary and metastatic tumors, rather than being completely overridden by tissue-specific differences. Consistent with this, matched primary tumor–liver metastasis pairs showed greater transcriptomic similarity than unmatched pairs (Figure 2A–C). These findings indicate that liver metastases retain substantial patient-specific molecular characteristics, highlighting the importance of paired analysis for identifying recurrent metastasis-associated transcriptional changes. At the same time, paired DEG and GO analyses identified recurrent transcriptional and biological differences between primary tumors and liver metastases (Figure 3A–C). Notably, liver metastases showed enrichment of both metastasis-related processes and pathways associated with liver physiology. Several biological processes enriched in liver metastases, including lipid and cholesterol metabolism, amino acid metabolism, and coagulation-related pathways, are also characteristic of normal liver tissue. Likewise, some biological processes enriched in primary tumors may be influenced by stromal or smooth-muscle components of the bowel wall. Previous studies showed that, during liver metastasis, colorectal cancer cells undergoing liver metastasis can acquire liver-associated transcriptional features [15], suggesting that adaptation to the hepatic microenvironment may contribute to such patterns. Nevertheless, an important consideration in interpreting these results is that bulk RNA sequencing reflects not only intrinsic alterations within tumor cells but also differences in the relative proportions of cell populations comprising the tumor microenvironment [16,17,18]. Therefore, the observed differences cannot be exclusively associated with tumor-intrinsic transcriptional reprogramming. Rather, our findings likely reflect a combination of tumor-cell-associated changes and differences in the tissue microenvironment between primary and metastatic sites.

GSEA further revealed changes in biological programs associated with EMT, angiogenesis, and hypoxia—processes long implicated in metastatic progression (Figure 4A). Specifically, EMT increases cell motility and invasive capacity, thereby promoting metastatic potential in colorectal cancer [19,20]. Hypoxia signaling drives metabolic reprogramming, enabling tumor cells to survive and adapt within the metastatic niche [21]. Angiogenesis supports the formation of new vasculature that sustains tumor growth as metastatic lesions establish within the hepatic microenvironment [22]. In this context, enrichment of EMT, angiogenesis, and hypoxia may be interpreted not only as activation of individual metastatic programs but also as part of a broader integrated metastatic state shaped by reciprocal interactions between tumor cells and the surrounding microenvironment [23,24]. Such a state may encompass coordinated changes in tumor-cell plasticity, extracellular matrix and stromal remodeling, vascular adaptation, and hypoxic responses that collectively support the survival, adaptation, and outgrowth of tumor cells within the hepatic niche [25,26]. Accordingly, these enriched biological programs may reflect the combined biological consequences of tumor-intrinsic alterations and microenvironmental remodeling during liver metastasis, rather than purely cell-intrinsic changes.

We next integrated multiple independent datasets to identify genes that were consistently altered across cohorts. Concordance between the overlap-based selection and Robust Rank Aggregation further strengthened the robustness of the candidate prioritization by reducing dependence on a single cutoff-based criterion and supporting the reproducibility of these genes across independent datasets. This supports the interpretation that the prioritized genes reflect recurrent metastasis-associated transcriptional features rather than cohort-specific or threshold-dependent findings. Several of the prioritized candidates are associated with liver, stromal, immune, vascular, or other tissue-related compartments. However, this does not necessarily reduce their biological relevance to metastasis, as several of these genes have independently been implicated in tumor progression, metastatic behavior, or remodeling of the metastatic niche. SPP1, for instance, has been linked to extracellular matrix remodeling, immune regulation, and metastatic processes across various cancer types, including colorectal cancer [27,28]. APOC1 and APOE, both involved in lipid metabolism, have recently been reported to contribute to tumor progression and remodeling of the tumor microenvironment [29,30,31]. In addition, CYP1B1 is known to be associated with cancer progression, metabolic regulatory changes, and invasive phenotypes [32,33]. FMO3 is associated with cholesterol metabolism [34], but recent evidence also implicates FMO3 in colorectal cancer cell proliferation, migration, invasion, and EMT-related phenotypes [34,35,36]. More broadly, metabolic reprogramming is increasingly recognized as an important component of cancer progression [37,38]. Thus, the recurrent increase in FMO3 observed in metastatic samples may reflect not only liver-associated expression but also a potential link to metabolic adaptation during metastatic progression. These previous observations provide biological plausibility for interpreting at least a subset of the recurrent expression changes as being related to metastatic progression or metastatic niche remodeling. Taken together, the recurrence of previously reported metastasis-related genes in our paired analysis and across independent validation cohorts supports the biological consistency of the identified transcriptional changes. However, these findings should not be interpreted to mean that each candidate independently drives metastasis. Instead, the collective recurrence of these genes suggests that they capture reproducible molecular features of the primary-to-liver-metastasis transition. Thus, the contribution of this study lies not solely in gene-level novelty, but in identifying a recurrent transcriptional pattern that emerges despite substantial interpatient heterogeneity.

Survival analysis was used as a complementary and exploratory assessment of the clinical relevance of the prioritized metastasis-associated genes across available colorectal cancer cohorts. While several cohorts assessed gene expression in primary colorectal tumors, GSE159216 provided survival information linked to colorectal liver metastasis samples. Because molecular features related to metastatic potential may already be detectable in the primary tumor, primary-tumor expression may still have potential biological relevance to later metastatic progression. Thus, these analyses explored whether candidate gene expression in either primary or metastatic tumor tissues was associated with clinical outcome, rather than establishing a uniform prognostic effect across tissue types.

One notable aspect of our study is the use of multiple independent transcriptomic cohorts for external validation. Because our analysis was based on a relatively small number of clinical samples, patient-specific or transcriptomic variation could have a disproportionate influence on the overall results [39]. To address this limitation, we compared the paired DEG results with multiple independent publicly available datasets and prioritized genes that showed consistent changes in the same direction across cohorts. This multi-cohort validation approach increases the likelihood that the identified candidate genes reflect molecular features more broadly associated with colorectal liver metastasis, rather than findings confined to our own dataset. In addition, the lack of reproducibility for some candidates in the qPCR analysis of the colorectal cancer cell model suggests that these expression changes may be more dependent on tissue-specific regulation or the tumor microenvironment. The HT29-M model was not intended to represent the full molecular diversity of colorectal cancer liver metastasis or to demonstrate that the identified transcriptional changes occur universally across colorectal cancer cells. Rather, it was used as a complementary experimental model to assess whether metastasis-associated changes identified in patient tissues could also be reproduced, at least in part, in an isolated colorectal cancer cell context. Thus, the concordant changes observed for some candidates support a potential tumor-cell-associated component, whereas the non-reproduced changes may be more strongly influenced by the surrounding tissue environment. Nevertheless, because this analysis was based on a single colorectal cancer cell line, further validation in additional colorectal cancer models will be required to assess the generalizability of these findings.

Our study has several limitations. First, the discovery cohort consisted of a relatively small number of paired clinical samples, and the generalizability of the findings should therefore be confirmed in larger paired cohorts. Second, the transcriptomic profiles were derived from bulk tissue, and the relative contributions of tumor cells and microenvironmental components cannot be fully resolved. Single-cell or spatial transcriptomic approaches will be important for further defining the cellular origins of the identified changes. Third, although the reproducibility of the prioritized candidates was supported across independent datasets and, for selected genes, in the HT29-M model, their functional contribution to metastatic progression remains to be established. Further functional studies are required to determine whether these genes directly contribute to metastatic progression or whether their altered expression arises as a consequence of changes within the metastatic microenvironment.

## 5. Conclusions

In conclusion, colorectal cancer liver metastasis was accompanied by recurrent transcriptomic changes shared across multiple patients despite substantial patient-specific molecular backgrounds. These findings suggest that metastasis may involve common biological processes that extend beyond patient-specific events. The reproducible molecular features identified here provide a framework for the future discovery of biomarkers and therapeutic targets for metastatic colorectal cancer.

## Data Availability

The RNA-sequencing data generated and analyzed in this study have been deposited in the NCBI GEO and are available under accession number GSE347316. The publicly available data analyzed in this study are openly available in the GEO database under accession numbers GSE14297, GSE213402, GSE50760, GSE49355, GSE39582, GSE17536, GSE19537, GSE159216, and GSE39084, and in The Cancer Genome Atlas (TCGA), including TCGA-COAD and TCGA-READ. Additional data supporting the findings of this study are available from the corresponding author upon reasonable request.

## Supplementary Materials

The following supporting information can be downloaded at: https://www.mdpi.com/article/10.3390/cimb48101033/s1.

## Abbreviations

The following abbreviations are used in this manuscript:

CRC	Colorectal cancer
CCLM	Colorectal cancer liver metastasis
DEGs	differentially expressed genes
GSEA	Gene Set Enrichment Analysis
TCGA	The Cancer Genome Atla
GEO	Gene Expression Omnibus
VST	Variance-stabilizing transformation
GO	Gene ontology
BP	Biological Process
HT29-P	HT29 parental cell
HT29-M	HT29-metastasis selected cell
qPCR	Quantitative real-time polymerase chain reaction
NES	Normalized enrichment scores
OS	Overall survival
DFS	Disease-free survival
DSS	Disease-specific survival
CSS	Cancer-specific survival
RFS	Recurrence-free survival
RRA	Robust Rank Aggregation
DW	Distilled water

## References

[1] Cañellas-Socias A., Sancho E., Batlle E. (2024). Mechanisms of metastatic colorectal cancer. Nat. Rev. Gastroenterol. Hepatol..

[2] Ni Y., Liang Y., Li M., Lin Y., Zou X., Han F., Cao J., Li L. (2023). The updates on metastatic mechanism and treatment of colorectal cancer. Pathol. Res. Pract..

[3] Ding Q., Chang C.J., Xie X., Xia W., Yang J.Y., Wang S.C., Wang Y., Xia J., Chen L., Cai C. (2011). APOBEC3G promotes liver metastasis in an orthotopic mouse model of colorectal cancer and predicts human hepatic metastasis. J. Clin. Investig..

[4] Tian B.W., Han C.L., Wang H.C., Yan L.J., Ding Z.N., Liu H., Mao X.C., Tian J.C., Xue J.S., Yang L.S. (2023). Effect of liver metastasis on the efficacy of immune checkpoint inhibitors in cancer patients: A systemic review and meta-analysis. Clin. Exp. Metastasis.

[5] Patel R.K., Rahman S., Schwantes I.R., Bartlett A., Eil R., Farsad K., Fowler K., Goodyear S.M., Hansen L., Kardosh A. (2023). Updated Management of Colorectal Cancer Liver Metastases: Scientific Advances Driving Modern Therapeutic Innovations. Cell Mol. Gastroenterol. Hepatol..

[6] Birrer D.L., Tschuor C., Reiner C., Fritsch R., Pfammatter T., Garcia Schüler H., Pavic M., De Oliveira M., Petrowsky H., Dutkowski P. (2021). Multimodal treatment strategies for colorectal liver metastases. Swiss Med. Wkly..

[7] Molinari C., Marisi G., Passardi A., Matteucci L., De Maio G., Ulivi P. (2018). Heterogeneity in Colorectal Cancer: A Challenge for Personalized Medicine?. Int. J. Mol. Sci..

[8] Blank A., Roberts D.E., Dawson H., Zlobec I., Lugli A. (2018). Tumor Heterogeneity in Primary Colorectal Cancer and Corresponding Metastases. Does the Apple Fall Far From the Tree?. Front. Med..

[9] Love M.I., Huber W., Anders S. (2014). Moderated estimation of fold change and dispersion for RNA-seq data with DESeq2. Genome Biol..

[10] Chen E.Y., Tan C.M., Kou Y., Duan Q., Wang Z., Meirelles G.V., Clark N.R., Ma’ayan A. (2013). Enrichr: Interactive and collaborative HTML5 gene list enrichment analysis tool. BMC Bioinform..

[11] Kuleshov M.V., Jones M.R., Rouillard A.D., Fernandez N.F., Duan Q., Wang Z., Koplev S., Jenkins S.L., Jagodnik K.M., Lachmann A. (2016). Enrichr: A comprehensive gene set enrichment analysis web server 2016 update. Nucleic Acids Res..

[12] Zhang Z., Liu S., Xu T., Chen N., Liu C., Yang H., Shi Y., Li Z., Feng X., Yao Y. (2025). Tumor suppressor SLC9A2 inhibits colorectal cancer metastasis and reverses immunotherapy resistance by suppressing angiogenesis. J. Exp. Clin. Cancer Res..

[13] Chen H., Zhai C., Xu X., Wang H., Han W., Shen J. (2023). Multilevel Heterogeneity of Colorectal Cancer Liver Metastasis. Cancers.

[14] Zhou H., Liu Z., Wang Y., Wen X., Amador E.H., Yuan L., Ran X., Xiong L., Ran Y., Chen W. (2022). Colorectal liver metastasis: Molecular mechanism and interventional therapy. Signal Transduct. Target. Ther..

[15] Teng S., Li Y.E., Yang M., Qi R., Huang Y., Wang Q., Zhang Y., Chen S., Li S., Lin K. (2020). Tissue-specific transcription reprogramming promotes liver metastasis of colorectal cancer. Cell Res..

[16] Yoshihara K., Shahmoradgoli M., Martínez E., Vegesna R., Kim H., Torres-Garcia W., Treviño V., Shen H., Laird P.W., Levine D.A. (2013). Inferring tumour purity and stromal and immune cell admixture from expression data. Nat. Commun..

[17] Aran D., Sirota M., Butte A.J. (2015). Systematic pan-cancer analysis of tumour purity. Nat. Commun..

[18] Jew B., Alvarez M., Rahmani E., Miao Z., Ko A., Garske K.M., Sul J.H., Pietiläinen K.H., Pajukanta P., Halperin E. (2020). Accurate estimation of cell composition in bulk expression through robust integration of single-cell information. Nat. Commun..

[19] Morgado-Diaz J.A., Wagner M.S., Sousa-Squiavinato A.C.M., de-Freitas-Junior J.C.M., de Araújo W.M., Tessmann J.W., Rocha M.R., Morgado-Diaz J.A. (2022). Epithelial-Mesenchymal Transition in Metastatic Colorectal Cancer. Gastrointestinal Cancers.

[20] Bocci F., Schneider-Stock R., Banerjee S. (2022). Editorial: Epithelial to Mesenchymal Plasticity in Colorectal Cancer. Front. Cell Dev. Biol..

[21] Hiraga T. (2018). Hypoxic Microenvironment and Metastatic Bone Disease. Int. J. Mol. Sci..

[22] Sun W. (2012). Angiogenesis in metastatic colorectal cancer and the benefits of targeted therapy. J. Hematol. Oncol..

[23] Yan T., Shi J. (2024). Angiogenesis and EMT regulators in the tumor microenvironment in lung cancer and immunotherapy. Front. Immunol..

[24] Quail D.F., Joyce J.A. (2013). Microenvironmental regulation of tumor progression and metastasis. Nat. Med..

[25] Hu P., Sun J., Lu J., Ge C., Sun H., Lv C. (2026). Navigating the Tumor Microenvironment in Colorectal Liver Metastasis: Barriers to Therapy and Emerging Opportunities. Oncol. Res..

[26] Chandra R., Karalis J.D., Liu C., Murimwa G.Z., Voth Park J., Heid C.A., Reznik S.I., Huang E., Minna J.D., Brekken R.A. (2021). The Colorectal Cancer Tumor Microenvironment and Its Impact on Liver and Lung Metastasis. Cancers.

[27] Liang X., Qin F., Yuan Z., Wu M., Zhang J., Liu X., Chen D. (2026). Colorectal cancer-derived osteopontin rewires macrophages into a pro-metastatic M2 state via the PI3K/AKT/CSF1-CSF1R axis. Cell Death Discov..

[28] Xu C., Sun L., Jiang C., Zhou H., Gu L., Liu Y., Xu Q. (2017). SPP1, analyzed by bioinformatics methods, promotes the metastasis in colorectal cancer by activating EMT pathway. Biomed. Pharmacother..

[29] Darwish N.M., Al-Hail M.K., Mohamed Y., Al Saady R., Mohsen S., Zar A., Al-Mansoori L., Pedersen S. (2023). The Role of Apolipoproteins in the Commonest Cancers: A Review. Cancers.

[30] Zhao Z., Zou S., Guan X., Wang M., Jiang Z., Liu Z., Li C., Lin H., Liu X., Yang R. (2018). Apolipoprotein E Overexpression Is Associated With Tumor Progression and Poor Survival in Colorectal Cancer. Front. Genet..

[31] He L., Shi M., Ren S., Zhang J., Tian Y., Yang X., Liu H. (2023). Jun-APOE-LRP1 axis promotes tumor metastasis in colorectal cancer. Biomol. Biomed..

[32] Jin L., Huang J., Guo L., Zhang B., Li Q., Li H., Yu M., Xie P., Yu Q., Chen Z. (2023). CYP1B1 promotes colorectal cancer liver metastasis by enhancing the growth of metastatic cancer cells via a fatty acids-dependent manner. J. Gastrointest. Oncol..

[33] Liu X., Ding J., Zong Q., Mao Y. (2025). B7-H3 enhances the malignant phenotype of colorectal cancer by activating the Wnt/β-catenin pathway mediated by CYP1B1. Tissue Cell.

[34] Schugar R.C., Brown J.M. (2015). Emerging roles of flavin monooxygenase 3 in cholesterol metabolism and atherosclerosis. Curr. Opin. Lipidol..

[35] Su R., Huang C., Jing Z., Qiu S., Shao L., Jiao T., Wang H., Li H. (2026). Targeting FMO3 suppresses the malignant phenotype of colorectal cancer: Evidence from bioinformatics to functional experiments. Future Sci. OA.

[36] Faubert B., Solmonson A., DeBerardinis R.J. (2020). Metabolic reprogramming and cancer progression. Science.

[37] Ohshima K., Morii E. (2021). Metabolic Reprogramming of Cancer Cells during Tumor Progression and Metastasis. Metabolites.

[38] Jiang W., Jin W.L., Xu A.M. (2024). Cholesterol metabolism in tumor microenvironment: Cancer hallmarks and therapeutic opportunities. Int. J. Biol. Sci..

[39] Michiels S., Koscielny S., Hill C. (2005). Prediction of cancer outcome with microarrays: A multiple random validation strategy. Lancet.

