# Supplementary material for: Paired Transcriptomic Profiling Identifies Shared Liver Metastasis-Associated Molecular Features in Colorectal Cancer
Source: Curr Issues Mol Biol. 2026 Oct 5;48(10):1033. doi: 10.3390/cimb48101033 (PMC13703309; doi:10.3390/cimb48101033)
Supplement: Supplementary file 1 [file cimb-48-01033-s001.zip › Figure S3.pdf]

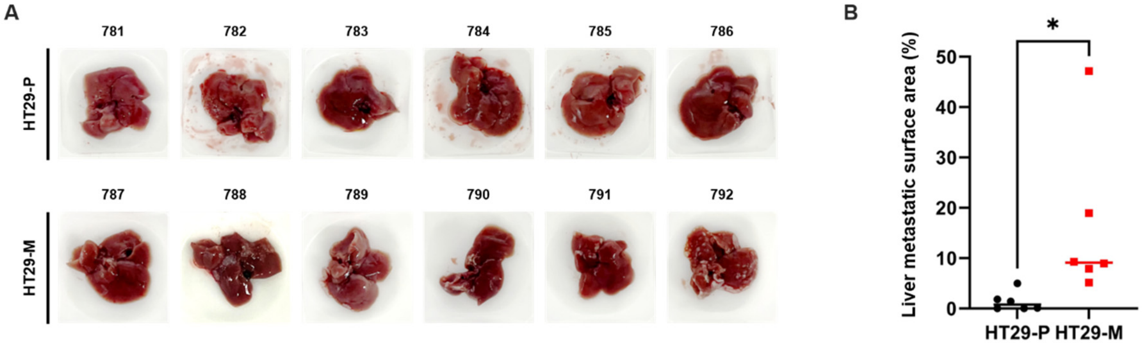

Figure S3. Liver metastasis in HT29-P and HT29-M mouse models.
(**A**) Liver images from mice injected with HT29-P or HT29-M cells. (**B**) Quantification of liver metastatic surface involvement, calculated as the metastatic area relative to the total liver surface area. Each point represents one mouse. *p < 0.05.
