# Supplementary material for: Paired Transcriptomic Profiling Identifies Shared Liver Metastasis-Associated Molecular Features in Colorectal Cancer
Source: Curr Issues Mol Biol. 2026 Oct 5;48(10):1033. doi: 10.3390/cimb48101033 (PMC13703309; doi:10.3390/cimb48101033)
Supplement: Supplementary file 1 [file cimb-48-01033-s001.zip › Figures S1 and S2.pdf]

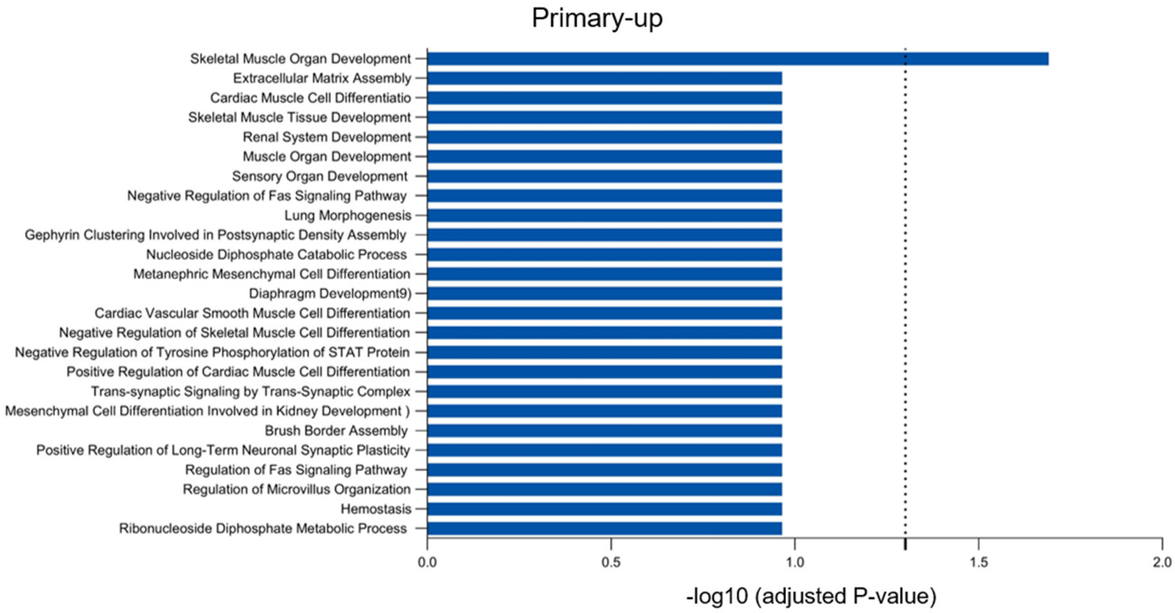

Figure S1. Gene Ontology biological process (GO-BP) enrichment analysis of primary-upregulated genes. Bars represent −log10 (adjusted P values) for enriched biological processes; the dashed line indicates an adjusted P value of 0.05.

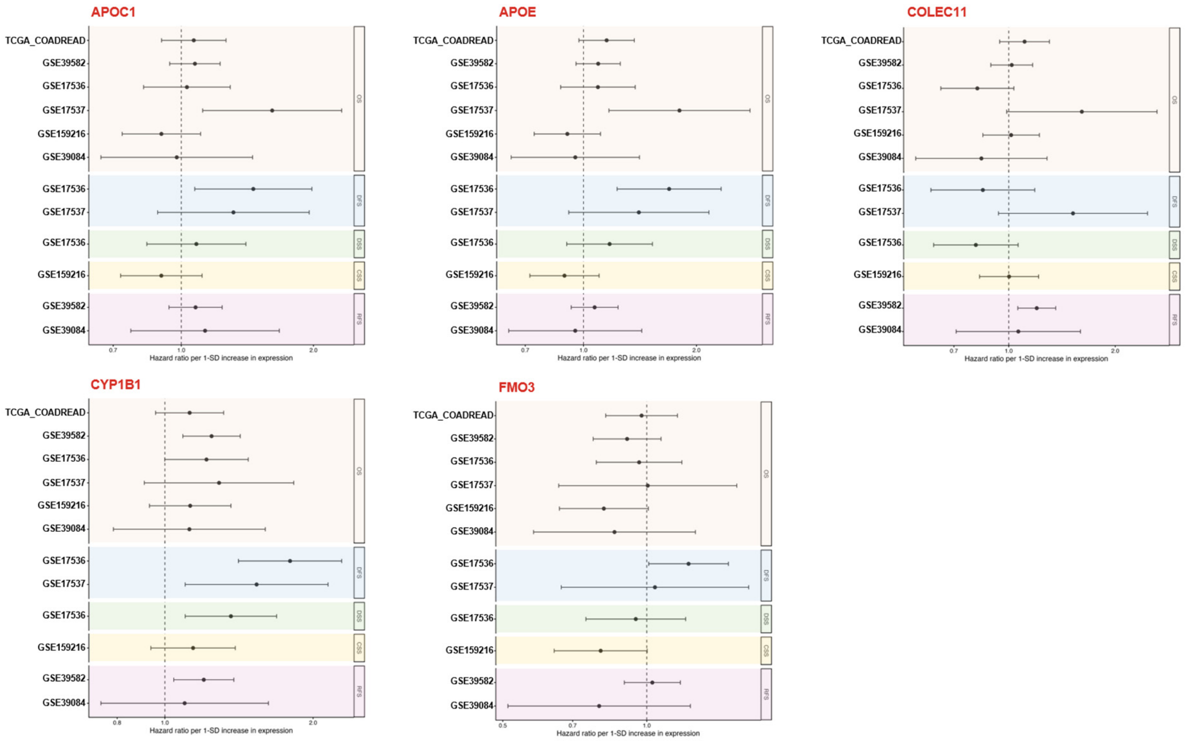

Figure S2. Forest plots showing the associations of APOC1, APOE, COLEC11, CYP1B1, and FMO3 expression in multiple datasets containing survival information. Hazard ratios (HRs) are presented per one-standard-deviation increase in gene expression, with horizontal lines indicating 95% confidence intervals. The dashed vertical line indicates HR = 1. Survival outcomes include overall survival (OS), disease-free survival (DFS), disease-specific survival (DSS), cancer-specific survival (CSS), and recurrence-free survival (RFS). The dashed vertical line indicates HR = 1.
